# Editorial: Structural and Computational Glycobiology – Immunity and Infection

**DOI:** 10.3389/fimmu.2015.00359

**Published:** 2015-07-14

**Authors:** Mark Agostino, Elizabeth Yuriev

**Affiliations:** ^1^CHIRI Biosciences and Curtin Institute for Computation, School of Biomedical Sciences, Curtin University, Perth, WA, Australia; ^2^Centre for Biomedical Research, Burnet Institute, Melbourne, VIC, Australia; ^3^Medicinal Chemistry, Monash Institute of Pharmaceutical Sciences, Monash University, Melbourne, VIC, Australia

**Keywords:** glycobiology, structural biology, infection, cancer immunotherapy, molecular modeling, molecular recognition, lectins, signaling

Historically deemed as the realm of the brave or the foolhardy, glycobiology has grown considerably as a discipline over the last 50 years. Carbohydrates, which were once considered to be mere “decorations” on proteins and lipid membranes, are increasingly demonstrated to afford specific roles in signaling and communication (1).

Although the rate of structures deposited into the Protein Data Bank continues to grow at an exponential rate, the characterization of new structures of carbohydrate–protein complexes is growing more modestly, still being very challenging and prone to errors (2). Computational methods are increasingly being pursued to provide structural insight into carbohydrate–protein interactions. The complex structure and high flexibility of carbohydrates, as well as difficulties associated with accurately computing binding energies for these interactions, present considerable challenges for the use of these methods in both understanding the carbohydrate–protein recognition and the structure-aided design of carbohydrate-based therapeutics. However, numerous computational approaches have been developed in recent years that address some of these issues (3–9). The Opinion piece in this Research Topic further highlights some computational resources that have been developed specifically for glycobiology (10).

Several carbohydrate classes, most notably gangliosides, Lewis antigens, and Thomsen– Friedenreich antigen, are of considerable interest for the development of cancer immunotherapeutics. Krengel and Bousquet (11) present a comprehensive review on the importance of gangliosides not only to cancer therapeutics but also their relevance for signaling and in mediating infection by pathogens, as well as how their structure and presentation on glycolipids and glycoproteins influences their function and potential to be exploited in therapeutics. Ahmed et al. (12) describe the use of molecular modeling to optimize framework regions of an anti-ganglioside antibody, resulting in the identification of a new construct with enhanced stability, antigen binding, and cytotoxic properties. Kieber-Emmons et al. (13) discuss the challenges and frontiers associated with the development of peptides as immunogenic mimics of carbohydrates, particularly focusing on mimics of tumor-associated carbohydrate antigens.

Despite considerable advances in the understanding of many aspects of glycobiology, several fundamental processes remain only partially understood. An excellent example of this is the structural basis of antibody recognition of the blood group antigens (A, B, H). Makeneni et al. (14) combine docking with a recently developed carbohydrate-specific scoring function and molecular dynamics simulation to demonstrate the structural basis of A vs. B specificity of an anti-A antibody. Lee et al. (15) performed LC-MS/MS-based glycomics and proteomics, combined with structural analyses, of a wide range of glycosylated proteins in order to understand the differences in the glycosylation of secreted cell surface and intracellular proteins. The study correlates the presence of specific *N*-glycan terminations with their subcellular location, providing insight into pathophysiological conditions caused by glycosylation disorders. Brockhausen (16) provides a comprehensive review detailing known glycosyltransferases with overlapping activities between bacteria and mammals. In many cases, similar catalytic mechanisms between bacterial and mammalian glycosyltransferases can be identified, despite limited sequence similarity.

Lectins, particularly C-type lectins, are of considerable importance for immunity, mediating cell–cell recognition, and representing potential targets for the development of therapeutics. Notable C-type lectins include DC-SIGN and the selectins, known for their roles in the progression of HIV and cancer, respectively. Richardson and Williams (17) review the discovery and characterization of the macrophage C-type lectin (MCL) and the macrophage-inducible C-type lectin (Mincle), their roles in initiating the immune response to infection, and the identification of activating ligands for these receptors. Aretz et al. (18) predict the druggability of a panel of C-type lectins, as well as perform fragment-based screening by nuclear magnetic resonance spectroscopy against DC-SIGN, langerin, and MCL. Their work highlights limitations in the application of computational methods to predict the druggability of this class of proteins.

The work presented in this Research Topic illustrates a small selection of the wide ranging research in this area and the considerable challenges associated with both understanding glycan function and targeting glycan interactions for the development of therapeutic agents.

## Conflict of Interest Statement

The authors declare that the research was conducted in the absence of any commercial or financial relationships that could be construed as a potential conflict of interest.
